# Inflammatory Markers as Predictors of Onset and Recovery of Olfactory and Gustatory Dysfunction in COVID-19 Patients

**DOI:** 10.7759/cureus.96003

**Published:** 2025-11-03

**Authors:** Burak Celik, Fatih Gul, Hacı Huseyin Dere

**Affiliations:** 1 Otolaryngology - Head and Neck Surgery, Ankara Bilkent City Hospital, Ankara, TUR; 2 Otolaryngology - Head and Neck Surgery, Lokman Hekim University, Faculty of Medicine, Ankara, TUR

**Keywords:** ageusia, chemosensory dysfunction, covid-19, loss of smell, loss of taste, post-covid-19, post-covid-19 anosmia

## Abstract

Background

Olfactory and gustatory dysfunctions are widely recognized as characteristic manifestations of COVID-19. However, the relationship between disease severity, inflammatory markers, and the recovery of chemosensory functions remains unclear, particularly in hospitalized patients. This study aimed to evaluate predictive hematological and radiological factors associated with the development and recovery of olfactory and gustatory dysfunction in COVID-19 patients.

Methodology

This prospective, observational study included 96 hospitalized patients diagnosed with COVID-19 via polymerase chain reaction testing between September 2020 and January 2021. A total of 53 patients with new-onset olfactory and/or gustatory dysfunction and 43 control subjects were selected through online randomization. Visual Analog Scale (VAS) scores were used to assess symptom severity and recovery. Hematological and inflammatory parameters, including neutrophil-to-lymphocyte ratio (NLR), corrected NLR, neutrophil-to-monocyte ratio (NMR), platelet-to-lymphocyte ratio (PLR), C-reactive protein (CRP), and procalcitonin levels, were analyzed. Thorax CT scores were used to determine disease severity. Follow-up evaluations were conducted by telephone at 90 days post-discharge to reassess VAS scores and recovery status.

Results

Olfactory loss was present in 42 (43.7%) patients, and taste loss in 44 (45.8%) patients. Olfactory dysfunction was significantly associated with age and higher thorax CT scores (p < 0.05). Procalcitonin and CRP levels were significantly associated with the development of both olfactory and gustatory dysfunction (p < 0.05). Lower NLR, d-NLR, NMR, and PLR values were strong predictors of recovery. At 90-day follow-up, 66.7% of patients with anosmia achieved complete recovery, 30.9% showed partial improvement, and 2.4% reported no recovery. All patients with taste impairment recovered (75% complete, 25% partial). Chemosensory dysfunction was more common in patients with milder disease severity.

Conclusions

Olfactory and gustatory dysfunctions are more prevalent in patients with milder COVID-19 and lower inflammatory burden. Elevated inflammatory markers and severe thoracic involvement were inversely associated with the development and recovery of chemosensory dysfunction. These findings suggest that systemic inflammation may play a detrimental role in neural recovery of chemosensory pathways and highlight the prognostic value of hematological markers in COVID-19 symptom monitoring.

## Introduction

Anosmia (complete loss of smell) and hyposmia (reduced sense of smell) are common symptoms associated with COVID-19 infection. Postviral anosmia is one of the main causes of olfactory dysfunction in adults, accounting for approximately 40% of cases [1]. Since the emergence of SARS-CoV-2, the number of individuals experiencing anosmia has markedly increased. The virus causes olfactory dysfunction primarily through the interaction of its spike (S) protein with angiotensin-converting enzyme 2 receptors located on the supporting (sustentacular) cells of the olfactory epithelium, leading to secondary impairment of olfactory neurons [2].

Anosmia or hyposmia has been reported as an early feature of COVID-19, with studies showing a prevalence ranging from 4% to 85%, depending on factors such as the viral variant, assessment methods, and study population [3]. Furthermore, anosmia has been identified as the initial symptom in over 26.6% of patients, and, in some cases, it may occur as the only manifestation of infection [4].

This study aims to evaluate the relationship between olfactory and gustatory dysfunction and laboratory markers in COVID-19 patients to clarify potential associations between sensory loss and disease severity.

## Materials and methods

Study population and severity classification of COVID-19

The study was conducted at Ankara City Hospital between September 1, 2020, and January 31, 2021, and included patients who had tested positive for COVID-19 via polymerase chain reaction test and were admitted to the Infectious Diseases service. The majority of the patients were classified as having moderate or severe COVID-19 based on thorax CT results, as determined by the guidelines established by the World Health Organization (WHO) [5]. The hospitalization, treatment, management, and discharge decisions for these patients were made in accordance with the guidelines established by the Ministry of Health [6]. Ethics committee approval was obtained from the Ethics Committee of Yıldırım Beyazıt University Faculty of Medicine (approval number: 43, dated 07/04/2021).

A detailed medical history was obtained for each patient upon admission, including information about any olfactory or gustatory disturbances, onset of symptoms, pre-existing conditions, and previous sinonasal surgeries. The severity of olfactory and/or gustatory disturbances was assessed using the Visual Analog Scale (VAS) during the patients’ hospital stay. Due to the risk of COVID-19 transmission, physical examinations were not performed.

COVID-19 classification according to symptoms

Of the 324 patients followed up between September 2020 and January 2021, 112 had new-onset loss of smell and/or taste. Of these 112 patients, 53 were selected through randomization to participate in the study, and 43 patients without olfactory/gustatory disturbances were selected as the control group. All participating patients provided informed consent. During the selection of the patient and control groups, online randomization was applied for both cohorts; however, no matching was performed based on comorbidities.

The VAS was standardized such that a score of 10 indicated normal smell/taste perception, 0 represented complete loss, and 5 corresponded to a 50% reduction in olfactory or gustatory function. Complete recovery was defined as a VAS score returning to 10, reflecting restoration of normal olfactory/gustatory function, whereas scores below 10 were categorized as partial recovery.

Patients who were under 18 or over 90 years of age; unable to communicate; had a history of malignancy, radiotherapy, or chemotherapy; were pregnant or lactating; had a history of nasal surgery, chronic rhinosinusitis, or any other condition that may affect olfactory function; had a history of psychiatric drug use; previous head trauma; or previous olfactory and/or gustatory disturbances were excluded from the study.

The study also included examination of blood tests such as whole blood, biochemistry, C-reactive protein (CRP), D-dimer, procalcitonin, international normalized ratio (INR), troponin, and inflammatory parameters such as neutrophil-to-lymphocyte ratio (NLR), corrected NLR, lymphocyte-to-monocyte ratio, neutrophil-to-monocyte ratio (NMR), platelet-to-lymphocyte ratio (PLR), and systemic inflammatory index, which were followed daily during the patient’s hospital stay, and the lowest, highest, and average values of these parameters were compared.

The thorax CT scans obtained during hospitalization were evaluated and scored by a single radiologist. The CT images were first evaluated for typical findings of COVID-19 pneumonia, as defined by consensus guidelines established by the North American Society of Radiology. The images were then classified as “no pneumonia,” “mild-severe involvement,” “moderate-severe involvement,” or “severe involvement” [7].

Finally, patients were contacted by phone an average of 90 days after discharge to inquire about any ongoing olfactory and gustatory disturbances and to reassess their VAS scores. The results of these follow-up inquiries were recorded.

Statistics analysis

The data collected in the study were presented in the form of mean ± standard deviation. The distribution of the data was determined using the Kolmogorov-Smirnov test. Continuous variables with a normal distribution were compared using the independent-sample t-test, while non-normally distributed variables were compared using the Mann-Whitney U test when appropriate. To compare the groups, a one-way analysis of variance was used. To identify the effect of independent variables between groups, binary logistic regression was performed. The statistical analyses and calculations were conducted using the SPSS Statistics version 27.0 software for Macintosh (IBM Corp., Armonk, NY, USA), and the level of statistical significance was set at p-values <0.05.

## Results

The study included 96 patients, 44 (45%) of whom were male and 52 (55%) were female. The mean age of the patients was 58.46 ± 21.03 years (range = 31-88 years). Of the 42 patients with olfactory loss, 18 (42%) were male and 24 (58%) were female. Similarly, 20 (46%) of the 44 patients with loss of taste were male and 24 (54%) were female. The control group, consisting of 43 individuals who did not experience olfactory or gustatory disturbances, was composed of 19 (44%) male and 24 (56%) female patients (Table 1).

**Table 1:** Sociodemographic characteristics in COVID-19 cases. LoS = loss of smell; LoT = loss of taste; LoST = loss of smell and taste

COVID-19 patients	Total (N = 96)	LoS (N = 42)	LoT (N = 44)	Without LoST (N = 43)	P-value	
					LoS	LoT
Age (year) (mean ± SD)	58.46 ± 21.03	56.62 ± 20.48	57.28 ± 19.73	60.57 ± 19.42	0.012	0.052
Male	55.26 ± 23.25	53.79 ± 21.15	54.53 ± 20.31	59.78 ± 20.61	-	
Female	60.84 ± 22.39	58.49 ± 21.83	59.02 ± 19.37	62.49 ± 19.27	-	
Sex (%)					0.36	0.43
Male	44 (45%)	18 (42%)	20 (46%)	19 (44%)	-	
Female	52 (55%)	24 (58%)	24 (54%)	24 (56%)	-	

The analysis revealed that age was a statistically significant factor in the development of olfactory loss (p < 0.05), with the age of onset for loss of taste being close to statistically significant (p = 0.052). The study did not find any statistically significant association between the presence of comorbidities and the development of olfactory or gustatory disturbances (Table 1).

The severity of the disease, as determined by thorax CT scores, was found to be a statistically significant factor in the development of olfactory and gustatory disturbances. Additionally, the minimum saturation values experienced by the patients during their hospitalization were found to be statistically significant in the development of olfactory loss (Table 2). The minimum and mean procalcitonin values were also found to be statistically significant in the development of olfactory loss (Table 3). Similarly, the mean procalcitonin and the maximum and mean CRP values were found to be statistically significant in the development of gustatory loss (Table 4).

**Table 2:** Relationship between thorax CT scores, saturation, and loss of smell and taste. LoS = loss of smell; LoT = loss of taste; LoST = loss of smell and taste

COVID-19 patients	Total (N = 96)	Without LoS (N = 54)	LoS (N = 42)	P-value	Without LoT (N = 52)	LoT (N = 44)	P-value	Without LoST (N = 43)
Thorax CT scores	10.63 ± 3.34	11.72 ± 3.62	9.23 ± 3.24	0.001	11.96 ± 3.66	9.06 ± 3.00	0.000	12.32 ± 3.53
Minimum saturation value	78.73 ± 5.58	76.50 ± 6.84	81.61 ± 4.53	0.000	76.03 ± 6.50	81.93 ± 4.73	0.000	75.09 ± 6.42

**Table 3:** Significant predictors of the development of olfactory loss. LoS = loss of smell

Predictive	N = 96	Values	P-value
Procalcitonin, minimum	Without LoS, N = 54	0.065 ± 0.084	0.02
	LoS, N = 42	0.030 ± 0.024	
Procalcitonin, maximum	Without LoS, N = 54	0.19 ± 0.276	0.06
	LoS, N = 42	0.108 ± 0.129	
Procalcitonin, mean	Without LoS, N = 54	0.116 ± 0.162	0.019
	LoS, N = 42	0.061 ± 0.046	

**Table 4:** Significant predictors of the development of loss of taste. LoT = loss of taste; CRP = C-reactive protein

Predictive	N = 96	Values	P-value
Procalcitonin, minimum	Without LoT, N = 52	0.063 ± 0.078	0.087
	LoT, N = 44	0.040 ± 0.048	
Procalcitonin, maximum	Without LoT, N = 52	0.193 ± 0.271	0.056
	LoT, N = 44	0.108 ± 0.150	
Procalcitonin, mean	Without LoT, N = 52	0.118 ± 0.156	0.023
	LoT, N = 44	0.061 ± 0.072	
CRP, minimum	Without LoT, N = 52	0.026 ± 0.032	0.063
	LoT, N = 44	0.015 ± 0.023	
CRP, maximum	Without LoT, N = 52	0.098 ± 0.083	0.013
	LoT, N = 44	0.062 ± 0.054	
CRP, mean	Without LoT, N = 52	0.059 ± 0.054	0.022
	LoT, N = 44	0.036 ± 0.039	

Furthermore, several inflammatory parameters, such as NLR (minimum-maximum and average), d-NLR (minimum-maximum and average), NMR (minimum-maximum and average), and PLR (minimum-maximum and average), were found to be statistically significant in the recovery of olfactory loss (Table 5). NLR (maximum and average), d-NLR (maximum and average), and NMR (minimum-maximum and average) were found to be statistically significant in the recovery of gustatory loss (Table 6).

**Table 5:** Significant predictive values of smell loss recovery. NLR = neutrophil-to-lymphocyte ratio; c_NLR = corrected neutrophil-to-lymphocyte ratio; NMR = neutrophil-to-monocyte ratio; PLR = platelet-to-lymphocyte ratio

Predictive	N = 42	Values	P-value
NLR, minimum	No recovery or partial recovery, N = 14	5.02 ± 3.51	0.003
	Complete recovery, N = 28	2.54 ± 1.52	
NLR, maximum	No recovery or partial recovery, N = 14	13.89 ± 11.51	0.006
	Complete recovery, N = 28	6.65 ± 4.75	
NLR, mean	No recovery or partial recovery, N = 14	8.66 ± 6.44	0.004
	Complete recovery, N = 28	4.26 ± 2.92	
c_NLR, minimum	No recovery or partial recovery, N = 14	3.19 ± 2	0.002
	Complete recovery, N = 28	1.73 ± 0.85	
c_NLR, maximum	No recovery or partial recovery, N = 14	7.77 ± 5.64	0.004
	Complete recovery, N = 28	4.07 ± 2.31	
c_NLR, mean	No recovery or partial recovery, N = 14	5.14 ± 3.4	0.002
	Complete recovery, N = 28	2.68 ± 1.35	
NMR, minimum	No recovery or partial recovery, N = 14	13.15 ± 8.14	0.008
	Complete recovery, N = 28	8.18 ± 3.44	
NMR, maximum	No recovery or partial recovery, N = 14	34.08 ± 25.91	0.006
	Complete recovery, N = 28	18.56 ± 8.19	
NMR, mean	No recovery or partial recovery, N = 14	21.8 ± 14.87	0.005
	Complete recovery, N = 28	12.7 ± 4.7	
PLR, minimum	No recovery or partial recovery, N = 14	254.35 ± 168.28	0.042
	Complete recovery, N = 28	172.42 ± 86.45	
PLR, maximum	No recovery or partial recovery, N = 14	516.51 ± 323.21	0.04
	Complete recovery, N = 28	344.44 ± 201.38	
PLR, mean	No recovery or partial recovery, N = 14	368.74 ± 227.87	0.044
	Complete recovery, N = 28	251.36 ± 137.42	

**Table 6:** Significant predictive values of taste loss recovery NLR = neutrophil-to-lymphocyte ratio; c_NLR = corrected neutrophil-to-lymphocyte ratio; NMR = neutrophil-to-monocyte ratio

Predictive	N = 44	Values	P-values
NLR, minimum	No recovery or partial recovery, N = 11	4.38 ± 3.27	0.097
	Complete recovery, N = 33	2.89 ± 2.25	
NLR, maximum	No recovery or partial recovery, N = 11	11.87 ± 11.37	0.027
	Complete recovery, N = 33	6.45 ± 4.51	
NLR, mean	No recovery or partial recovery, N = 11	7.44 ± 6.09	0.043
	Complete recovery, N = 33	4.38 ± 3.4	
c_NLR, minimum	No recovery or partial recovery, N = 11	2.83 ± 1.65	0.11
	Complete recovery, N = 33	1.99 ± 1.42	
c_NLR, maximum	No recovery or partial recovery, N = 11	6.9 ± 5.88	0.027
	Complete recovery, N = 33	4.11 ± 2.26	
c_NLR, mean	No recovery or partial recovery, N = 11	4.63 ± 3.44	0.03
	Complete recovery, N = 33	2.85 ± 1.76	
NMR, minimum	No recovery or partial recovery, N = 11	13.38 ± 9.11	0.049
	Complete recovery, N = 33	9.07 ± 4.78	
NMR, maximum	No recovery or partial recovery, N = 11	33.97 ± 28.23	0.035
	Complete recovery, N = 33	20.57 ± 12.59	
NMR, mean	No recovery or partial recovery, N = 11	21.84 ± 16.41	0.028
	Complete recovery, N = 33	13.77 ± 7.17	

Among the study population, there were patients presenting with isolated olfactory dysfunction and isolated gustatory dysfunction; however, the majority exhibited a combined loss of both smell and taste.

After the 90-day follow-up period, patients were contacted via telephone and their VAS scores were reassessed. Among the 42 patients who had reported olfactory dysfunction, 28 (66.7%) achieved complete recovery, 13 (30.9%) demonstrated partial improvement, and one (2.4%) reported no recovery. In contrast, all 44 patients with gustatory dysfunction showed improvement; 33 (75%) achieved complete recovery, and 11 (25%) exhibited partial recovery.

In summary, the study found that certain demographic and clinical factors, such as age and thorax CT scores, as well as certain inflammatory markers, were associated with the development and improvement of olfactory and gustatory disturbances in COVID-19 patients.

## Discussion

Anosmia or hyposmia is a well-established characteristic symptom of COVID-19. In fact, it is considered a key diagnostic indicator for COVID-19 by the Centers for Disease Control and Prevention in the United States [7]. A meta-analysis published in 2020 estimated the prevalence of olfactory dysfunction in COVID-19 patients to be 52.7% [3]. Studies conducted among hospitalized patients have reported the prevalence of olfactory dysfunction to be 25% [8] and 34% [9]. In our study, we found the prevalence of olfactory dysfunction to be 34.5%. Similar to other studies, our study found that patients with olfactory dysfunction were younger, female, and had fewer comorbidities [3,10,11]. Bénézit et al. reported the prevalence of olfactory and gustatory dysfunction in COVID-19 patients to be 75% and 92.65%, respectively [12]. Due to the presence of comorbidities and the severity of COVID-19 in patients who participated in our study, we found a lower prevalence of olfactory and gustatory dysfunction. We believe that in patients with milder forms of the disease, who are able to recover from the disease at home, the prevalence of these dysfunctions is higher.

According to WHO reports, as of April 25, 2022, over 510 million COVID-19 cases have been reported, with 47.55% being male and 52.45% being female (WHO, 2022). While studies have shown that the distribution of COVID-19 cases among genders is similar, it has been highlighted that the clinical course of the disease is worse in male patients [13]. In our study, 45% of the patients were male, 55% were female, and the average age was 58.46 ± 21.03 years.

Previous studies have shown that individuals with loss of smell are less associated with severe illness compared to those without loss of smell [14]. Talavera et al. found that individuals with loss of smell were younger, had fewer comorbidities, and were primarily female, and that the mortality rate of those with anosmia was lower. This study also showed that individuals with anosmia had higher hemoglobin and lymphocyte counts, as well as lower D-dimer and CRP values [8]. Our study also found that laboratory values were similar in individuals with loss of smell and/or taste.

The presence of chronic lung diseases is considered a poor prognostic indicator in COVID-19 patients [15]. Studies have shown that pro-inflammatory cytokines such as interleukin (IL)-6, CRP, IL-10, lactate dehydrogenase, and tumor necrosis factor-alpha are associated with increased oxygenation requirements and the development of acute respiratory distress syndrome, dialysis, and ventilation in COVID-19 patients [16]. Additionally, research has revealed that individuals with olfactory loss have lower levels of IL-6, indicating a less severe disease course [17]. Our findings support the idea that high levels of CRP and procalcitonin are indicative of severe disease, and the likelihood of developing loss of smell and/or taste in these patients is low. Furthermore, we found no significant association between D-dimer and the development or improvement of smell or taste loss. Additionally, high NLR and CRP can be evaluated as indicators of severe disease, and the probability of developing a loss of smell or taste in severe patients is low.

In a study, Cheng et al. observed that there was a statistically significant difference in the distribution of age between mild-to-moderate COVID-19 patients who were hospitalized and those who had olfactory loss or did not have olfactory loss. However, no significant differences were found between the two groups in terms of comorbidity, gender, and laboratory findings [18]. In our study, we found that age was statistically significant in relation to loss of smell and close to statistically significant in relation to loss of taste (p = 0.012 for loss of smell, p = 0.052 for loss of taste). In contrast to the findings of Cheng et al., our study found a significant difference in the development and/or recovery of some inflammatory values in relation to loss of smell and taste [18].

In the study conducted by Hopkins et al., the recovery rate of olfactory loss was in the 80s in several weeks of follow-ups [19]. In contrast, Babaei et al. found the recovery rate of anosmia to be 88.5% and 93.2% in four-week and eight-week periods, respectively [20]. In our study, we followed up with our patients for an average of 12 weeks and found the recovery rate to be 97% for loss of smell and 100% for loss of taste. Only one of our patients, who experienced only loss of smell, did not show any improvement during the 12-week follow-up.

In their study, Printza et al. did not find a difference in the prevalence of loss of smell and taste between mild-to-moderate COVID-19 patients [21]. In our study, we found that the prevalence of loss of smell and taste decreased as the severity of the disease increased.

Von Bartheld et al. and Yan et al. reported that the prevalence of chemosensory deficiency is higher in patients who do not require hospitalization [22,23]. As our study was limited only to hospitalized patients, we could not comment on this topic, but we showed that as the severity of the disease increased, the prevalence of chemosensory deficiency decreased in hospitalized patients.

In a study conducted by Moein et al. on hospitalized patients with a high prevalence of olfactory dysfunction, there was no significant correlation between the severity of COVID-19 and olfactory dysfunction [24]. However, in our study, we demonstrated a correlation between disease severity and the development of olfactory and/or gustatory loss.

Limitations

This study has several limitations that should be acknowledged. First, it was conducted at a single center and included only hospitalized patients, which may limit the generalizability of the findings to individuals with mild or asymptomatic disease who were managed in outpatient settings. Second, olfactory and gustatory dysfunctions were assessed subjectively using the VAS rather than objective psychophysical tests such as the Sniffin’ Sticks or University of Pennsylvania Smell Identification Test, which may have introduced reporting bias. Third, comorbidities were not matched between the study and control groups during randomization, which may have acted as potential confounding variables. Fourth, inflammatory markers and hematologic indices were evaluated only during hospitalization; longitudinal biomarker measurements following discharge were not performed. Finally, neuroimaging methods or olfactory cleft endoscopy were not utilized to investigate structural or neural mechanisms underlying chemosensory impairment. Future multicenter studies with larger sample sizes, objective olfactory/gustatory testing, and long-term follow-up are warranted to validate and extend these findings.

## Conclusions

In this study, olfactory and gustatory dysfunctions were found to be more prevalent among COVID-19 patients with milder disease severity and lower inflammatory burden. Thoracic CT involvement and elevated inflammatory markers such as CRP, procalcitonin, NLR, and NMR were inversely associated with the development of chemosensory impairment, suggesting that severe systemic inflammation may predominantly affect lower respiratory tract involvement rather than the olfactory epithelium or neural pathways. Importantly, chemosensory recovery was observed in the majority of affected patients within 90 days, and lower inflammatory indices were significant predictors of complete recovery. These findings indicate that inflammatory status may play a pivotal role not only in the pathogenesis but also in the prognosis of COVID-19-related olfactory and gustatory dysfunction. Early identification and longitudinal monitoring of these patients may provide prognostic value, and future studies incorporating objective olfactory assessment and neurobiological analysis are warranted to better elucidate underlying mechanisms and guide targeted therapeutic strategies.
